# Holding a Handle for Balance during Continuous Postural Perturbations—Immediate and Transitionary Effects on Whole Body Posture

**DOI:** 10.3389/fnhum.2016.00486

**Published:** 2016-09-26

**Authors:** Jernej Čamernik, Zrinka Potocanac, Luka Peternel, Jan Babič

**Affiliations:** ^1^Department for Automation, Biocybernetics and Robotics, Jožef Stefan InstituteLjubljana, Slovenia; ^2^Jožef Stefan International Postgraduate SchoolLjubljana, Slovenia; ^3^HRI2 Laboratory, Department of Advanced Robotics, Istituto Italiano di TecnologiaGenoa, Italy

**Keywords:** falls, handle, grasping, postural balance, balance recovery

## Abstract

When balance is exposed to perturbations, hand contacts are often used to assist postural control. We investigated the immediate and the transitionary effects of supportive hand contacts during continuous anteroposterior perturbations of stance by automated waist-pulls. Ten young adults were perturbed for 5 min and required to maintain balance by holding to a stationary, shoulder-high handle and following its removal. Center of pressure (COP) displacement, hip, knee and ankle angles, leg and trunk muscle activity and handle contact forces were acquired. The analysis of results show that COP excursions are significantly smaller when the subjects utilize supportive hand contact and that the displacement of COP is strongly correlated to the perturbation force and significantly larger in the anterior than posterior direction. Regression analysis of hand forces revealed that subjects utilized the hand support significantly more during the posterior than anterior perturbations. Moreover, kinematical analysis showed that utilization of supportive hand contacts alter posture of the whole body and that postural readjustments after the release of the handle, occur at different time scales in the hip, knee and ankle joints. Overall, our findings show that supportive hand contacts are efficiently used for balance control during continuous postural perturbations and that utilization of a handle has significant immediate and transitionary effects on whole body posture.

## Introduction

With aging society, falls are becoming an increasingly large problem. A large proportion of falls occur due to the improper weight shifts (Robinovitch et al., 2013) and impaired postural control is a landmark of aging (Maki and McIlroy, 2006; Mansfield and Maki, 2009). When postural control is impaired, handrails, canes and handles are often used to assist maintaining balance by providing additional supportive contacts with the environment. This indicates that holding onto a physical aid is beneficial for postural control.

With respect to the use of hand contacts for postural control, one of the widely investigated phenomena is “light touch” (Jeka, 1997; Krishnamoorthy et al., 2002). These light, fingertip contacts with stationary objects can extend the base of support (Bateni and Maki, 2005) and provide an additional sensory input, which helps individuals to better position them in space (Jeka, 1997). Such sensory information improves postural control in quiet standing by reducing the amplitude of center of pressure (COP) movement (Jeka, 1997; Johannsen et al., 2007; Kouzaki and Masani, 2008; Wing et al., 2011).

On the other hand, in case of perturbed balance reaching arm movements with the aim to grasp for a nearby object is a widely utilized change-in-support strategy (Maki and McIlroy, 2006). Such hand contacts provide mechanical support in addition to the sensory augmentation of the light touch and thus offer a better stabilizing potential in the presence of perturbations (Maki and McIlroy, 1997). Specifically, holding onto a handle increases the base of support of a standing individual and enables a person to generate necessary hand forces to better counteract the perturbations (Babič et al., 2014; Sarraf et al., 2014). A recent study by Babič et al. (2014) showed that the location of the supporting hand contact is important to maximize its stabilizing potential and that the peak forces exerted at the handle during the support surface perturbations are related to the location of the handle.

Aforementioned studies were based on the discrete perturbations of balance which predominantly evoke feedback postural responses. A major component of such responses is comprised of motor actions that are related to various sensorimotor reflexes and to a lesser extent to the feed-forward components of the postural control (Mergner, 2010). Moreover, the discrete perturbations evoke reach-to-grasp arm movements even when the perturbations are so light that they do not physically disturb postural balance (McIlroy and Maki, 1995; Corbeil et al., 2013). In contrast to the discrete perturbations of balance, perturbations that continuously disturb postural balance evoke both feedback and feed-forward components of motor action and in this sense offer a complementary insight into the postural control (Dietz et al., 1993; Schmid et al., 2011).

The remaining question is what is the role of hand contact during continuous perturbations? Therefore, the aim of this article is to study situations where balance of an individual is challenged by continuous postural perturbations and to investigate the role of supportive hand contact in counteracting postural perturbations. Specifically, our goal was to investigate the immediate and the transitionary effects of a supportive hand contact on postural control of an individual whose balance is challenged by continuous anteroposterior perturbations of stance.

Our hypothesis is that a supportive hand contact has a significant influence on postural balance by reducing the COP excursion during the perturbation and that the utilization of the hand contact is more prominent for postural perturbations in the backward direction which are more threatening than the perturbations in the forward direction. Moreover, we hypothesize that utilization of the additional hand support not only alters posture of the human body while the hand is in contact with the environment but also after the release of the handle. To effectively address these hypotheses, we developed an experimental framework where we continuously perturbed postural balance and investigated the relationships between the perturbation force and the COP displacement, kinematical parameters of the human body, and the forces exerted by the supportive hand.

## Materials and Methods

### Participants

Thirteen healthy right-handed young adults participated in this study after giving their written consent. Data of three subjects were excluded from the analyses due to technical problems during acquisition, therefore we used the data of ten subjects (average age = 22.3 years, SD = 2.2 years, average height 179.2 cm, SD = 5.9 cm and average weight = 76.9 kg, SD = 8.2 kg). The experimental procedures conformed to the latest revision of the Declaration of Helsinki and were approved by the Slovenian National Medical Ethics Committee (No. 112/06/13).

### Measurement Protocol

**Figure 1:**
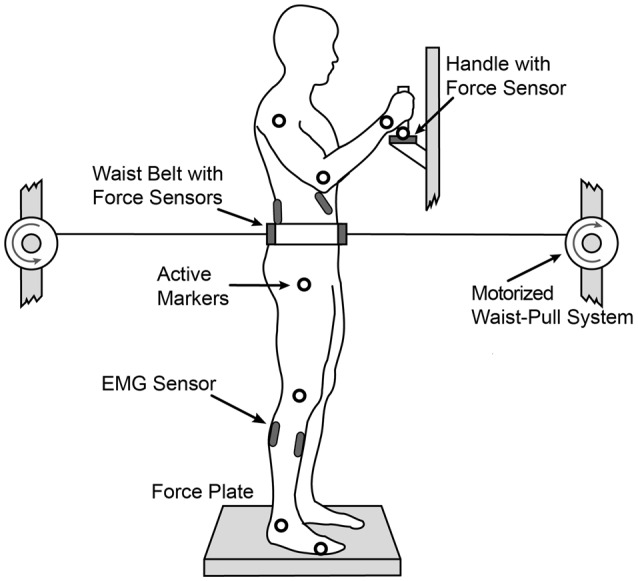
**Experimental setup.** The subject is standing on a force plate, wearing a waist belt connected to the motorized waist-pull system which generated translational force perturbations in the anterior-posterior direction using a band-filtered white noise signal constructed to emulate mild, daily life perturbations.

**Figure 2:**
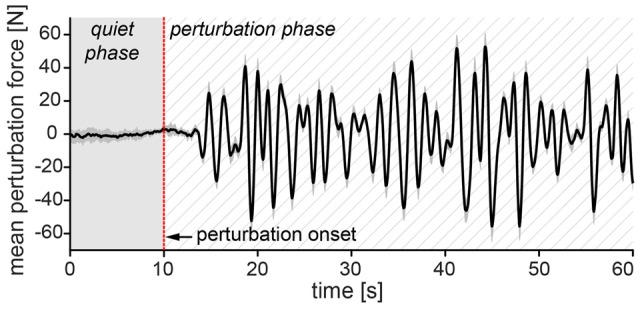
**Perturbation signal sample.** Solid black curve represents first 60 s of mean perturbation signal from all 10 subjects with ±1 standard error of the mean (gray shade around the curve). In the first 10 s of each trial the perturbation value was at 0 N—quiet phase. After 10 s (perturbation onset), the perturbation gradually increased with alternated direction and amplitude—perturbation phase. Positive values of mean perturbation force represent forces in anterior direction and negative values represent forces in posterior direction. The perturbation signal was identical in both trials.

Subjects were asked to step on a force plate, stand straight with the feet placed at hip width and look straight ahead. They were required to keep upright posture and maintain balance without making any unnecessary corrective steps while their balance was continuously perturbed in anteroposterior direction by a motorized waist-pull system (Peternel and Babič, 2013) as depicted in Figure 1. During the experiment, the subjects were not allowed to change their base of support. We marked their individual standing position on the force plate prior to the start of the experiment which was used as a reference for foot position during the experiment. This ensured that the different stance width would not affect subjects’ balance since it has been shown by Bingham and Ting (2013) that active torque at ankle and hip joints scale with stance width. To emulate mild, daily life perturbations such as those during riding on buses, subways and trains (Graaf and van Weperen, 1997) the motorized waist-pull system perturbed the subjects using a band-pass filtered white noise signal (0.25–1.00 Hz) with the maximal perturbation force of 11% of the subjects’ body weight (Figure 2).

The experiment consisted of two consecutive 5 min trials of different standing conditions: balancing while holding to a handle [*with-handle (WH)*] and without holding to a handle [*no-handle (NH)*]. First, the subjects were exposed to 5 min of perturbations in the *WH* trial. In the *WH* trial, subjects held onto a stationary handle (diameter = 3.2 cm, length = 12 cm) positioned at shoulder height with their right hand. After 5 min the perturbation stopped, subjects released the handle and folded their arms across their chest. Then, on average less than 60 s later, the second trial of 5 min of perturbation started (*NH* trial). In the *NH* trial, subjects were standing with their arms folded across their chest. In both trials, subjects were instructed to look straight ahead at all times at a fixed point positioned at the subject’s eyelevel and 3 m in front of the experimental setup. To induce response adaptations (Van Ooteghem et al., 2008; Schmid et al., 2011), the subjects were allowed to familiarize with the experimental procedures prior to the main experimental trials.

Kinetic data were collected using a force plate (9281CA, Kistler Instrumente AG, Winterthur, Switzerland) under the subjects’ feet and a 3-axis force sensor (45E15A, JR3, Woodland, CA, USA) on the handle, both at 1000 samples/s.

Kinematic data were collected at a sampling rate of 100 samples/s using a contactless motion capture system (3D Investigator, Northern Digital Inc., Waterloo, ON, Canada) consisting of a 3 × 3 camera array. Nine active markers were placed at the apparent axis of rotation of the fifth metatarsophalangeal, ankle, knee, hip, shoulder, elbow and wrist joints on the subject’s right side as well as at the base of the platform and the handle.

Anteroposterior displacement of the subject’s COP was derived from the force plate data. In the first 10 s of each trial, the subjects stood quietly (holding a handle with right hand in the first trial, or both arms folded across their chest in the second trial) and no perturbation was applied at the waist (see Figure 2 for reference regarding perturbation signal). The mean COP position from this time period (we refer to this as *quiet phase*) served as a baseline for calculation of COP excursions in the anterior and posterior direction in the following perturbations (*perturbation phase*).

Handle forces were calculated by considering the torques of the lever (distance from force sensor to the middle of the subject’s hand on the handle). Kinematic data were low pass filtered (zero lag, 2nd order Butterworth filter with a cut-off frequency 20 Hz). Ankle angle was calculated as the angle between the foot (line connecting the fifth metatarsal and ankle) and the shank (line connecting the ankle and the knee), knee angle as the angle between the shank and thigh (line connecting the knee and the hip), and hip as the angle between the thigh and torso (line connecting the hip and the shoulder). To evaluate the adaptation effects of releasing the handle in the *NH* trial, an exponential fit of group average joint angles was calculated (Franklin et al., 2003). Adaptation was considered as final once the given joint angle reached the plateau defined by three time constants of the fitted exponential decay function, i.e., once the fitted exponential decay function fell to 5% of its starting value (Honeine et al., 2015; Assländer and Peterka, 2016).

Electromyographical (EMG) electrodes were placed on the right leg (TA, Tibialis Anterior; GA, Gastrocnemius Lateralis; and trunk (MF, Multifidus; OE, Obliques Externus) muscles and their activity was measured using Biometrics DataLOG MW8X at a sampling rate of 1000 samples/s. Preparation of the skin and positioning of the electrodes was performed according to the SENIAM protocol (Hermens et al., 2000). Before the start of the experiment, subjects performed three maximal voluntary contractions (MVC) against resistance of each of the measured muscles. MVC’s were used in EMG post-processing for normalization, to establish a common ground when comparing data between subjects. All EMG signals were band-pass filtered (zero lag, 2nd order Butterworth filter with cut-off frequencies of 20 and 450 Hz), full-wave rectified and low pass filtered (zero lag, 2nd order Butterworth algorithm, 10 Hz cut-off frequency). Finally, EMG signals were normalized with respect to the MVCs and integrated over time (iEMG) to express the magnitude of muscle activity.

We divided COP excursions and contact forces exerted on the handle in two data sets based on their direction—anterior and posterior. For each data set, average values from all 10 subjects were calculated and used in the statistical analysis.

Average hip, knee and ankle angles over the 5 min for each subject were calculated and used for statistical analysis.

Differences between COP displacement in the anterior and posterior directions and subject average joint angles were analyzed using paired samples *t*-tests. Differences between the *WH* and *NH* trials in subject average COP displacements were analyzed for the anterior and posterior directions separately, using a paired samples *t*-test. The relationship between group average COP excursion and the magnitude of the perturbation and between group average perturbation magnitude and the exerted handle contact force was analyzed using separate linear correlations for anterior and posterior directions. All statistical analyses were performed using SPSS 21 Inc., Chicago, IL, USA at *α* = 0.05. Effect size (*d*) was calculated using standard Cohen’s equation (Cohen, 1988).

## Results

### Supportive Hand Contact has Significant Influence on Postural Balance by Reducing COP Excursion during Perturbation

**Figure 3:**
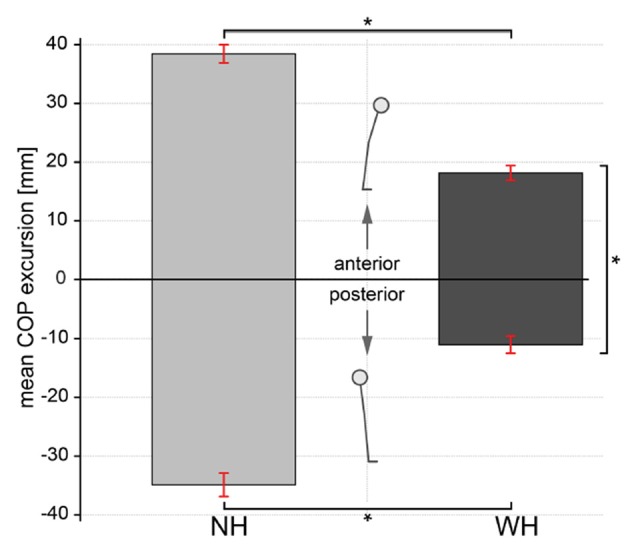
**Comparison of center of pressure (COP) excursion between trials when subjects either used additional hand contact or not.** Bars represent mean COP excursion during no-handle (NH) and with-handle (WH) trials for the anterior (positive) and posterior (negative) directions. Error bars indicate ±1 standard error of the mean. Statistically significant differences are indicated (*p ≤ 0.02).

The diagram in Figure 3 shows the comparison of mean COP excursion between the conditions when the subjects counteracted postural perturbations without using the additional hand contact (NH) and when they did use the handle (WH). Paired samples *t*-test showed significant effect in reducing the mean COP excursion when the subjects were holding to the handle compared to when they did not hold to the handle. The differences of COP excursion were significantly larger both in the anterior direction (difference of 20.3 mm, *t*_(9)_ = 7.78, *p* = 0.001, *d* = −4.15) and posterior direction (difference of 23.9 mm, *t*_(9)_ = −11.09, *p* = 0.001, *d* = −3.8).

### Utilization of Hand Contact is more Prominent for Postural Perturbations in Backward Direction than for Perturbations in Forward Direction

In both, *NH* and *WH* trials, the COP excursion was larger in the anterior direction (mean ± SE: *NH* 38.5 ± 1.6 mm, *WH* 18.2 ± 1.2 mm) compared to the posterior (mean ± SE: *NH* −34.9 ± 2 mm, *WH* −11.0 ± 1.5 mm), but this difference was significant only for the *WH* trial (*t*_(9)_ = 2.81, *p* = 0.02, *d* = 1.52).

**Figure 4:**
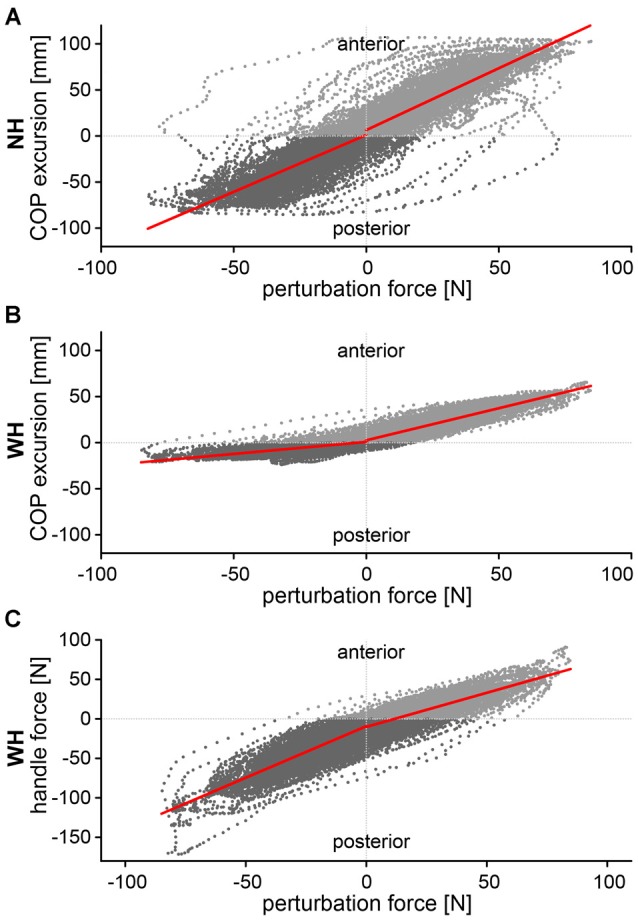
**Correlations between perturbation force and (A) COP excursion in NH trial, (B) COP excursion in WH trial and (C) handle force in the WH trial.** Correlations were calculated separately for the anterior (positive) and posterior (negative) direction.

We further assessed the effects of utilizing the additional hand contact, and the direction and intensity of perturbation on the maximal COP displacement. The diagrams in Figure 4 show correlations between the perturbation force and the group average COP excursion during *NH* and *WH* trials. Additionally, the correlation between the perturbation force and the handle force is shown for the *WH* trials. The group average COP excursion was strongly correlated with perturbation force in both posterior (*r*_p_ = 0.77 and *r*_p_ = 0.67) and anterior (*r*_a_ = 0.82 and *r*_a_ = 0.89) directions in the *NH* and *WH* trials, respectively (all *p* < 0.001). Similarly, the forces that subjects applied on the handle was also strongly correlated with the perturbation force (Figure 4C) in both anterior (*r*_p_ = 0.85, *p* < 0.001) and posterior directions (*r*_a_ = 0.81, *p* < 0.001). Moreover, the slope of the regression line is significantly larger for perturbations in the posterior direction (*k*_p_ = 1.3), compared to the perturbations in the anterior direction (*k*_a_ = 0.86).

### Supportive Hand Contact Affects Whole Body Posture

**Figure 5:**
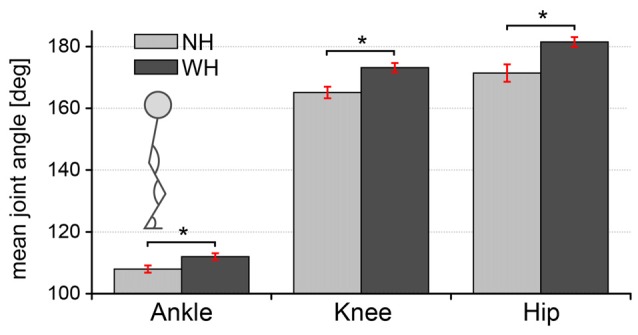
**Comparison of body posture between trials when subjects either used additional hand contact or not.** Bars represent mean ankle, knee and hip joint angles during NH and WH trials. Error bars indicate ±1 standard error of the mean. Statistically significant differences are indicated (*p ≤ 0.02).

To investigate how the additional hand contact affects the body posture during the perturbations, we compared the mean values of ankle, knee and hip joint angles between the conditions when the subjects counteracted perturbations without the additional hand contact (NH) and when they did use the handle (WH). The comparison is shown in the diagram in Figure 5.

Multiple paired samples *t*-tests showed significant effect of the hand contact on all three observed joint angles. Specifically, mean joint angles were significantly lower during the *NH* trials compared to the *WH* trials. Differences were the largest in the knee (mean ± SE: 165.1 ± 1.9° for NH, 173.2 ± 1.5° for WH, *t*_(9)_ = −6.70, *p* < 0.001, *d* = 1.4), followed by the hip (mean ± SE: 171.4 ± 2.8° for NH, 181.5 ± 1.6° WH, *t*_(9)_ = −6.68, *p* < 0.001, *d =* 1.1) and the ankle (mean ± SE: 108 ± 1.2° for NH, 112 ± 1.1° WH, *t*_(9)_ = −5.67, *p* < 0.001, *d* = 1.1).

### Utilization of Hand Contact has Non-Uniform Transitionary Effect on Whole Body Posture after Release of Handle

**Figure 6:**
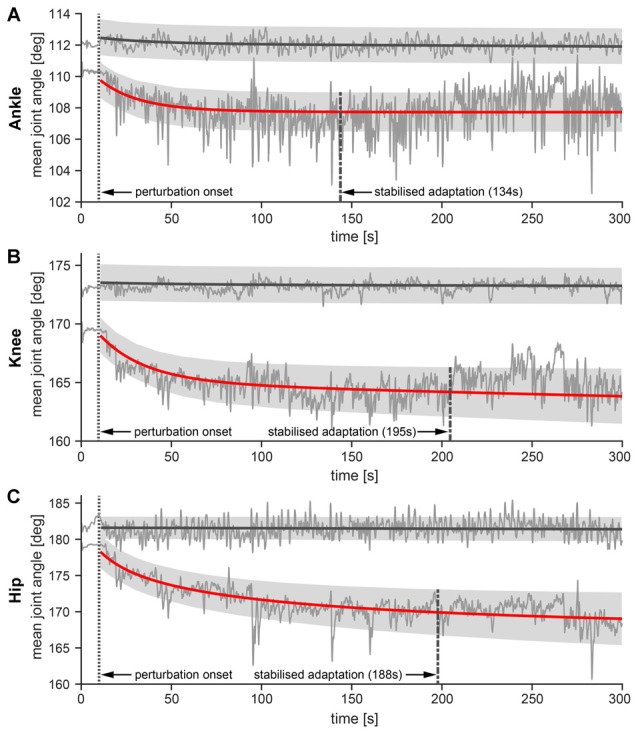
**Ankle (A) knee (B) and hip (C) angles over the time course of the perturbation.** Thin solid lines represent mean joint angles from all 10 subjects during NH and WH trials. Thick solid lines represent exponential curve fit denoting adaptation of joint angles in the NH (red color) and WH trials (gray color), while shaded areas represent ±1 standard error of the mean of the exponential decay curve. Mean *R*^2^ value for the exponential decay curves for ankle joint in the NH trial was mean ± SE: 0.27 ± 0.06, for the knee joint mean ± SE: 0.31 ± 0.08 and for the hip joint mean ± SE: 0.61 ± 0.08. The dotted vertical lines represent perturbation onset while the dashed vertical lines indicate the mean time of stabilized changes in the joint angles after perturbation onset.

To investigate the effect of supportive hand contact on the body posture, an exponential curve was fitted to the group average ankle, knee and hip joint angles calculated during the *NH* trial that immediately followed the *WH* trial (Figure 6).

Exponential fits revealed that postural readjustments after the release of the handle did not occur simultaneously throughout the body. Instead, the readjustments occurred at different time scales in the hip, knee and ankle joints. Specifically, joint angles stabilized first in the ankle (mean ± SE: 133 ± 103.5 s after perturbation onset), followed by the hip (mean ± SE: 188 ± 90.8 s after the perturbation onset) and finally in the knee (mean ± SE: 195 ± 92.5 s after the perturbation onset). However, a paired-samples *t*-test did not show statistically significant difference between any of the compared pairs due to the high variability of data.

### Analysis of Muscle Activity

**Figure 7:**
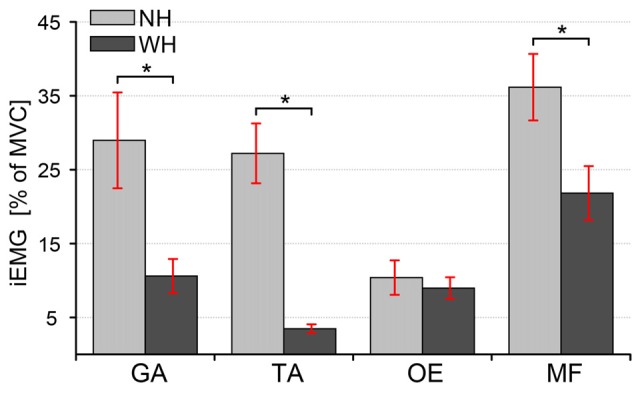
**Comparison of integrated Electromyographical (EMG) between trials when subjects either used additional hand contact or not.** Bars represent iEMG of four muscles (GA, Gastrocnemius Lateralis; TA, Tibialis Anterior; OE, Obliques Externus; MF, Multifidus) during NH and WH trials. Error bars indicate ±1 standard error of the mean. Statistically significant differences are indicated (*p ≤ 0.02).

Muscle activity was significantly lower during the WH trial than during the NH condition both for the leg muscles (GA *t*_(9)_ = 3.57, *p* = 0.04, *d* = −0.89; TA *t*_(9)_ = 6.41, *p* = 0.002, *d* = −23 1.85) and one of the trunk muscles (MF *t*_(9)_ = 6.5, *p* = 0.001, *d* = −1.01), as can be seen in Figure 7. Leg muscle activity was 18.4 ± 4.9% lower in the GA (mean ± SE: NH: 28.9 ± 6.5% MVC, WH: 10.6 ± 2.3% MVC) and for 23.7 ± 3.5% in the TA (mean ± SE: NH: 27.2 ± 4% MVC, WH: 3.47 ± 1.88% MVC), while the trunk muscle activity was 14.3 ± 2.1% lower in the MF (mean ± SE: NH: 36.2 ± 4.5% MVC, WH: 21.8 ± 3.7% MVC), but no significant change was observed in the OE (mean ± SE: NH: 10.4 ± 7.4% MVC, WH: 8.97 ± 4.63% MVC).

## Discussion

We investigated how subjects used an additional hand support (handle) to maintain upright posture when exposed to mild and continuous perturbations elicited by waist-pull apparatus in the anteroposterior directions. The use of handle reduced the destabilizing effect of the applied perturbation by reducing the excursions of COP. These results are in line with previous studies that investigated light-touch contacts. Those studies found a reduction of COP excursions during bipedal stance with (Johannsen et al., 2007; Hausbeck et al., 2009) and without externally applied perturbations (Jeka, 1997; Krishnamoorthy et al., 2002; Kouzaki and Masani, 2008). However, in our case the handle compensated for a significant load and served more than just a light-touch contact. The mean force in the handle was over 24 N (mean ± SE: 24.5 ± 7.9 N) where in other light-touch studies, contact forces usually did not exceed 3 N (Krishnamoorthy et al., 2002; Johannsen et al., 2007; Kouzaki and Masani, 2008). Comparison between measured mean handle force and mean perturbation force, which was ~20 N (mean ± SE: 19.5 ± 1.7 N), indicates that a significant portion of perturbation on postural stability was counterbalanced by the hand.

COP excursions were strongly correlated with the perturbation force in both directions, indicating the perturbations were effective, albeit mild. When holding a handle, the excursions of the COP were larger in the anterior than in posterior direction and less correlated with the perturbation force. Due to the specifics of our design, i.e., the use of a continuous perturbation, it was impossible to investigate pure feedback postural responses to the specific direction of the perturbation. However, asymmetry in COP excursion might to be due to a differential use of the handle, indicated by a steeper slope of the regression line between the perturbation force and the forces exerted on the handle for the posterior direction. This indicates that subjects have utilized the handle more when they counteracted the posterior COP excursions. This may be related to a more threatening situation due to the inability of the subjects to see (look) behind them, as the directions to the subjects were to look straight ahead at all times, and due to smaller stability margin in the posterior direction (Pai and Patton, 1997; Hof et al., 2005). This finding is in line with our previous study using a similar handle location and discrete perturbations caused by support platform translations, in which COP excursions were larger in the anterior direction (Babič et al., 2014).

Using a handle for balance support was beneficial, since it resulted in less displacement of COP and a smaller deviation from the neutral posture, as evident by the average joint angles. Measured joint angles in *WH* condition stayed closer to the neutral anatomic position than joint angles in the *NH* condition. Additionally, leg and trunk muscle activity was also significantly lower during the *WH* trial compared to the *NH* trial. This is consistent with the decreased leg muscle activity reported previously, when subjects had to hold (Cordo and Nashner, 1982) or touch (Sozzi et al., 2012) a surrounding object. Unlike other muscles, we found no decrease of muscle activity in the OE, which controls the rotation of the torso (Ng et al., 2001). The unilateral hand support might have caused a rotation of the trunk, which the subjects had to counteract by the OE activity. Prior to experiments we instructed subjects to use the handle in any way they prefer. Overall reduced muscle activity in legs and trunk and increased activity in arm muscles in case of using handle indicate that a portion of significant perturbation load was shifted from legs/trunk to arms. The same can be confirmed by high measured handle forces and lesser COP excursions in case of *WH* trial. The use of hand contact to compensate a significant portion of perturbation, even though it could be counteracted solely by legs/trunk, might be preferred since legs in a stance already have to compensate the load of the body mass (Bateni and Maki, 2005; Mergner, 2010).

Finally, when subjects had to release the handle for balance control they prepared for the more difficult *NH* condition even before the perturbation onset, as evident from differences in the starting joint angles. When the perturbation began this preparation was even more pronounced and joint angles changed further. These postural readjustments appear to occur at different time scales in the hip, knee and ankle joints, however that was not statistically confirmed. The subjects bended their ankle, knee and hip joints which resulted in a more flexed leg and lower hip position. Hip position can serve as an indication of the COM position and lowering of the COM could facilitate balance control (Rosker et al., 2011). Hence, these changes indicate feed-forward preparation to ease control of balance when expecting more challenging conditions, i.e., in the absence of handle. Along the same line, anticipation of the upcoming perturbations was also reported to cause changes in kinematics during quiet stance (Santos et al., 2010), walking (Pijnappels et al., 2001), recovery stepping (Pater et al., 2015) and tripping (Potocanac et al., 2014).

## Author Contributions

JC, LP and JB designed the study. JC and LP performed the experiments. JC, LP, ZP and JB analyzed the data and wrote the manuscript.

## Funding

The work presented in this article was supported by the European Community Framework Programme 7 through the CoDyCo project, contract no. 600716.

## Conflict of Interest Statement

The authors declare that the research was conducted in the absence of any commercial or financial relationships that could be construed as a potential conflict of interest.
